# How clinicians experience a simulated antiretroviral therapy adherence exercise: A qualitative study

**DOI:** 10.4102/phcfm.v10i1.1836

**Published:** 2018-10-25

**Authors:** Justin G. Engelbrecht, Fidele K. Mukinda, Beryl Green, Donald Skinner

**Affiliations:** 1 Division Health Systems and Public Health, Stellenbosch University, South Africa; 2 School of Public Health, University of the Western Cape, South Africa; 3 Department of Paediatrics and Child Health, Stellenbosch University, South Africa; 4 HIV, AIDS, STDs and TB Research Programme, Human Sciences Research Council, South Africa

## Abstract

**Background:**

With the shift of paediatric antiretroviral therapy (ART) from tertiary to primary health care, there has been a need to train clinicians working in primary health care facilities to support adherence to treatment. An adherence simulation exercise was included in a course on paediatric human immunodeficiency virus (HIV) and tuberculosis (TB) to stimulate health care providers’ awareness and generate empathy of complex paediatric adherence practices.

**Aim:**

The aim of this study was to describe the experience of clinicians completing the simulation exercise and to assess whether enhancing their empathy with patients and treatment supporters would improve their perceived clinical and counselling skills.

**Setting:**

The study was conducted at the Faculty of Medicine and Health Sciences, Stellenbosch University, and a guesthouse in Cape Town.

**Methods:**

The adherence module used blended learning methodology consisting of face-to-face contact sessions and distance learning. A qualitative thematic approach was used to understand the participant experiences through focus-group discussions and semi-structured interviews.

**Results:**

Three thematic clusters emerged, namely, experiences of the simulated exercise, patient–provider relationships and adherence strategies. Their experiences were both positive and challenging, especially when a ‘caregiver and/or treatment supporter’ scenario encouraged participants to reflect on their own relationships with their patients. Clinicians had also considered how empathy fits into their scope of responsibilities. Text messaging and adherence counselling strategies were identified.

**Conclusion:**

Simulated learning activities have the potential to create awareness of relationships between clinicians and their patients and generate ideas and discussion that could lead to improvements in clinical practice, and adherence promotion strategies.

## Introduction

Considerable progress has been made over the past 10 years in the scale-up of paediatric antiretroviral (ARV) therapy (ART) in resource-limited settings.^1^ Globally, by the end of 2016, approximately 43% of the estimated 2.1 million children living with the human immunodeficiency virus (HIV) were receiving ART.^2^ Improving access to paediatric ART in these settings is a constant challenge because of difficulties in diagnosis, complex ART formulations, and the limited skills and capacity of health care providers to initiate children on ART.^2^

The South African Department of Health ART guidelines (2010) have increasingly shifted paediatric ART delivery to the primary health care (PHC) level, and to nursing staff. This shift, while pragmatic, has placed a greater burden on PHC services. PHC requires capacity, skills, infrastructure and new strategies to address this shift.^3,4^ In addition, PHC staff are frequently not confident about caring for young HIV-positive children.^5^ ART requires sustained adherence (> 95%) to achieve optimal outcomes. In paediatric patients, ART adherence represents a significant challenge for children, their caregivers, and health care providers.^6^ Children rely on a caregiver, who may themselves be infected, to administer medication. Syrups are difficult to measure, and frequently bad tasting. These challenges may be aggravated in resource-limited settings.^7^ It is, therefore, critical that health care providers understand the factors that influence adherence in HIV-positive children.^8,6,9^

There are few training initiatives designed to ensure that health care providers understand the logistical and psychosocial challenges of taking ARVs once or twice daily.^10^ A simulated paediatric exercise conducted amongst clinic staff at a paediatric clinic in Swaziland found that most achieved poorer adherence than actual patients and identified similar barriers to adherence commonly reported by caregivers.^8^ An understanding of such challenges is important, if health care providers are to effectively discuss adherence strategies and plans with paediatric patients and their caregivers.^10^

Training initiatives, such as simulation exercises, may improve patient care by allowing clinicians to become better trained, without putting their patients at risk. These initiatives allow protected time for reflection debriefing, where learning often takes place.^9^ Research also suggests that simulation-based team training can improve quality of care and reduce patient mortality.^11^

South to South (S2S) Programme for Comprehensive Family HIV Care and Treatment is situated within the Department of Paediatrics and Child Health in the Faculty of Medicine and Health Sciences, at Stellenbosch University, South Africa.^12^ South to South addresses capacity-building at different levels of the health system, in order to improve maternal and child HIV and TB health outcomes. This capacity-building model is a multilevel, integrated intervention to improve the ability of individuals, organisations and system of the National Department of Health (NDOH), to implement scale-up and to institutionalise innovations to improve patient outcomes.^13^ S2S developed a Paediatric and Adolescent HIV/TB Care and Management short course, which aimed to capacitate health care providers in paediatric HIV and TB. Paediatric Adherence is a module within the course. A simulated paediatric adherence exercise, based on the Swaziland study, was included in this module, giving participants an opportunity to simulate caregiver and treatment supporter roles. The exercise was included to stimulate health care providers’ awareness and generate empathy of complex paediatric adherence practices. This awareness should assist health care providers to understand the context of caregivers, improving care. For the purpose of this paper, we will define empathy as the capacity to be sensitive to and to understand the feelings, thoughts and experiences of their patients who have to provide the doses to their children.^13^

The aim of this study was both to describe the experience of health care providers completing a simulation exercise and to assess whether enhancing their empathy with patients and treatment supporters would improve their perceived clinical and counselling skills.

## Research methods and design

### Study design

A qualitative thematic design was used to understand participants’ experiences of a simulated adherence activity through focus-group discussions (FGDs) and semi-structured interviews.

### Setting

The study was conducted at the Faculty of Medicine and Health Sciences, Stellenbosch University, Tygerberg Campus, and a guesthouse in Bellville, Cape Town, from March to October 2015.

### Study population, sample size and sampling

All the participants for the first three S2S Paediatric and Adolescent HIV/TB Care and Management short courses, facilitated in 2015, were included in the study. To be eligible, the participants had to be a doctor or integrated management of childhood illnesses (IMCI) nurse, involved in paediatric HIV management who had previously completed adult ART training. The final sample size was determined by data saturation.

Random allocation was used to select caregivers and treatment supporters for each training course. Each assigned caregiver was paired up with an assigned treatment supporter. Each course was anticipated to have 10 participants; however, the first course only had nine because of one cancellation. Because of the odd number of participants in this group, one caregiver had two supporters. The debriefing discussion at the end of the exercise doubled as a FGD of each of the three courses.

## Intervention

The Paediatric and Adolescent HIV/TB Care and Management short course uses a blended learning approach, consisting of face-to-face contact sessions and distance learning to support knowledge translation. The first contact week focused on content and was followed by three months of distance learning, which focused on case discussions, assignments in practical application and readings. The course concludes with a second contact week, focused on assignment feedback and content. The simulation exercise was facilitated during the first contact week of the course. The S2S facilitator allocated to participants the roles of a ‘caregiver’ or ‘treatment supporter’. All participants attended a group adherence session, similar to that offered to caregivers of paediatric clients initiating ART, including a review of the importance of adherence, the individual ARVs in the regimen, potential adverse effects, the components of successful adherence, and the consequences of poor adherence. Participants received simulated mixtures of first-line ARVs representing abacavir syrup 6 mL twice daily, lamivudine syrup 6 mL twice daily, and lopinavir/ritonavir syrup 2 mL twice daily. Water and food colourants were used to represent the ARVs. They were also given a syringe, adaptor cap and instructions. A colour-coding system, an innovative tool developed by the Western Cape Department of Health to enhance the caregiver’s confidence in administering paediatric liquid ARVs, was used to label the containers and syringes at the desired dosages.^14^ The participants were requested to strictly adhere to the appropriate schedule, administering the liquid into the sink. Treatment supporters were requested to remind the caregivers to administer the placebo drug. On the final day of the first contact week, the participants returned the remaining drugs, adherence was measured, and a debriefing discussion was conducted.

### Data collection

The FGDs conducted by the facilitator and lead researcher were audio-recorded and lasted for approximately 45 min. The lead researcher had completed a 6-month course in qualitative research and was confident in conducting interviews. Semi-structured interviews were conducted during the second contact week because they would have had the 3-month period to implement what they had learnt during the exercise. These semi-structured interviews included only those designated as caregivers, as they had more responsibility in administering the placebos. The semi-structured interviews lasted approximately 30 min, were audio-recorded and conducted in English. Nine interviews were completed to generate enough data for analysis. Only three FGDs and nine semi-structured interviews were conducted as the authors felt that data saturation was reached. For this reason, there was no need to conduct semi-structured interviews with FGD3 (see Table 1).

**TABLE 1:** Outline of course (2015).

Course	FGD (1st contact week)	Semi-structured interviews (second contact week)
1st course (March to June)	9 participants	4 interviews
2nd course (May to August)	10 participants	5 interviews
3rd course (July to October)	10 participants	0 interviews

FGD, focus-group discussion.

## Data analysis

The process of analysis was to generate meaning from the experiences of participants undertaking this exercise. Audiotapes were transcribed by a research assistant and checked by the first author for accuracy. Any additional notes documenting the interview context, participant emotions and body language were added to the transcripts. The transcripts and audio recordings were read and listened to until the author achieved an adequate level of data emersion, to ascertain patterns of thought. Sets of themes were identified by the material in the transcriptions, study objectives and researcher’s knowledge, and grouped together into clusters according to conceptual similarities.

The analysis was a continual process, and clusters were altered as data were collected and new themes emerged. Atlas.ti.7.1.6 was used to facilitate the analysis. This allowed for themes and codes to be changed accordingly.^15^ Transcripts were analysed according to relevant literature, as well as theoretical perspectives using a thematic analysis approach.

## Ethical consideration

The participants joined a training programme and were required to participate in all activities planned to reach the training objectives, including this simulation. Participation in the study was done with the written consent of the participants. Obtaining consent could have been seen as a form of coercion, as participants may have felt that by not participating they would not be able to complete the course. Every effort was therefore made to ensure that the study did not negatively impact the learning of the participants, and they were assured that declining to participate in, or withdrawing from the research interviews, would not compromise their learning. Other members of the training team reinforced this. Ethical clearance was sought from the Stellenbosch University Research Ethics Committee (S15/02/021) and permission from South to South (S2S) management. The research was not conducted at a government institution or the workplace of the participants; therefore, no permission was needed from their employers. No identifiers were used in the transcripts of the interviews. Data were stored in a password-protected computer. Recordings of the focus group discussions and semi-structured interviews were destroyed at the completion of the study. The transcripts and informed consent forms are locked away in a cupboard in the S2S offices and stored for five years.

## Results

This analysis describes responses to a simulated exercise done during a paediatric care training event. The three FGDs were denoted as FGD1, FGD2 and FGD3. The interviews with specific caregivers were described in terms of their profession (nurse [N] or doctor [D]) and age in years and gender (Male [M] or Female [F]). Table 2 describes participant demographics.

**TABLE 2:** Focus-group discussion participants.

FGD number	Number of participants	Age range	Median	Profession	*n*	Province	*n*
FGD 1	9	31–58 years	44	Doctors	3	Eastern Cape	4
	-	-	-	Nurses	6	Free state	4
	-	-	-	-	-	Western Cape	1
FGD 2	10	24–58 years	41	Doctors	3	Eastern Cape	6
	-	-	-	Nurse	7	Western Cape	2
	-	-	-	-	-	Limpopo	1
	-	-	-	-	-	Free State	1
FGD 3	10	31–61 years	46	Nurses	10	Mpumalanga	5
	-	-	-	-	-	Eastern Cape	2
	-	-	-	-	-	Limpopo	1
	-	-	-	-	-	Gauteng	1
	-	-	-	-	-	Free State	1

FGD, focus-group discussion.

The themes used for the analysis are presented in their clusters in Table 3.

**TABLE 3:** List of clusters and associated themes.

Experiences of the simulated exercise	Patient–provider relationship	Adherence strategies
Excitement Anxiety Stigma Disclosure Social life interruption Forgetfulness Daily schedule Practical difficulties	Improved relationship Respect Decision-making Empathy	Colour-coding Technological interventions Treatment supporter Counselling skills

### Experience of the simulated exercise

Participants reflected on both positive and negative experiences. Some felt anxious, while others were excited to simulate the caregiver and supporter role:

‘Yes. For me, on the first day, it was kind of exciting. I was actually looking forward to seven o’ clock, waiting to come and give the medication. I was not stressed.’ (FGD1, N, 50, F))

For two participants, the experience was emotional (caregiver and treatment supporter) as they identified with their own stigmatisation at work by their colleagues, because of their diabetes and hypertension, to stigma experienced by clients with HIV. These participants developed empathy with HIV-infected parents.

‘This exercise has made me realise about my own stigmatisation … as my colleagues at work stigmatise me since I am currently a diabetic and hypertension … bring my tablets to work.’ (FGD3, N, 49, F)

A participant (caregiver) from FGD2 related that she experienced a form of inadvertent disclosure during the exercise because of people seeing her medication at the guesthouse, or while shopping. This was interesting as assumptions were being made, based on what people had seen. All participants mentioned that lack of disclosure, and stigma, was still an ongoing problem at their settings, and that the exercise had warranted an urgent response to put measures in place to address this issue.

‘How will I carry this treatment … big bottles … and it will be obvious, during treatment time … for everybody to see … That’s where the issue of disclosure comes.’ (FGD2, N, 49, F))

The participants identified and experienced several barriers to adherence, also commonly reported by clients. These included the logistical challenges of taking medication and handling the bottles and syringes. The impact on daily routine and trying to keep to the exact time to administer the drug emerged during the reflective period following the exercise:

‘It messes up the routine, we as health care workers, we need to understand. It messes up your routine. Sometimes you are busy with something and you compensate for the treatment you didn’t administer yesterday.’ (FGD3, N, 48, F)

‘Changing the times … I would apply something. At the clinic, everybody’s talking about eight o clock. I don’t know if it’s the rule … we never ask if it suits the person.’ (FGD1, N, 41, F)

Even though all the participants were experienced clinicians, some shared that they had never handled bottles and syringes when managing their own patients. There was a sense of frustration amongst some of the participants when handling syringes and bottles, as they often spilled the liquid. Participants had experienced anxiety and doubt in their own ability to perform this task. They were not aware of the practical challenges that their patients experience when administering liquid formulations:

‘Tell him that it was not an easy exercise to do. I was anxious. So, I had to be on my feet. Firstly, my buddy phoned me, and I remember … It’s not easy, even though it’s marked.’ (FGD3, N, 50, F)

‘Theory is good … but when you come to a practical situation … that is when you get those hiccups. I have spilt a lot of this medication because I was struggling with the syringe of which I am not used to.’ (FGD2, N, 42, F)

### Patient–provider relationship

The shared experiences highlighted the importance of a patient–provider relationship, leading the participants to change their own relationships with their patients. A doctor made an explicit connection between this exercise and her relationship with her own patients in the clinic:

‘Experience has opened my mind and I think I am now more understanding of my patients … so it is life changing in a way.’ (D, 31, F)

Another doctor reported that she was more confident to provide adherence counselling, and clinically manage her patients, which made her a better-equipped health care professional:

‘I am a better doctor for that and I have changed the way I speak and handle patients now.’ (D, 29, F)

Participants were able to recognise their own limitations as health care providers through the exercise. Before this exercise, some participants were more interested in imposing their ideas on their patients regarding adherence, rather than asking for their patient’s input. They realised that being a good health care provider meant involving the patient in decision-making, which ultimately improves the patient–provider relationship. There needs to be mutual decision-making by both the caregiver and the child, when embarking on a treatment regimen:

‘That she as a health care professional was being respected by mothers and they are able to identify problems and solve them as well. It is a two-way relationship that involves a mutual decision-making effort.’ (FGD3, N, 44, F)

Interestingly, participants had mentioned that if the exercise had been extended, their empathy levels would have increased. This was emphasised by participants returning after the 3-month distance component. Many had felt that they had developed more empathy for their patients:

‘I think if it was any longer, I would have had more empathy for the patients.’ (D, 29, F)

### Adherence strategies

Many adherence strategies were identified by participants, and all realised the importance of assisting caregivers and children to be adherent. These strategies either were implemented or were going to be implemented at their facilities. Colour-coding of syringes and bottles, introduced as a tool to improve adherence during the exercise, was adopted by most participants for their own facilities to assist the caregivers to measure the correct dosage of medication.^15^

Participants were given the opportunity to experience having a ‘treatment supporter’, to remind them to administer the medication. This experience reminded them that treatment support of their own patients back at their facilities was lacking. One participant enquired why mothers of children were not being supported using a buddy system. Some participants promised to initiate this strategy as soon as they returned to their health care facilities:

‘Sometimes the caregiver doesn’t have treatment buddies at all. They just have the caregiver and the child, and that’s all. Even when you ask “Who can be your buddy?”, you’ll find that she has no one. They do fine, but … at the first time, you realise that, they needed support.’ (FGD2, N, 50, F)

The treatment supporters and caregivers used various adherence strategies to communicate. The cell phone technology was central to this. WhatsApp, an instant messaging system, was used by most treatment supporters to remind their caregivers to administer the drugs. Once the caregiver had administered the placebo drug, they would use this instant messaging service to send pictures of the administration to the treatment supporter:

‘I had it on my phone to remind me.’ (FGD1, N, 45, F)

‘I like to see the picture and prove to me, that she withdrew the amount.’ (FGD2, N, 43, F)

Most participants agreed that intensive adherence counselling was an important strategy in adherence, and some realised that they were providing this service inadequately in their settings. Many had also realised that using closed-ended questions while managing their patients at their facilities was not ideal. They realised that approaching their patients in a non-threatening way, with open-ended questioning, would elicit more information. This was a novel insight. The exercise gave them an opportunity to explore their own values and attitudes towards their patients:

‘We always use close-ended questions. More than open-ended, whereby now you are finalising things for the client.’ (FGD2, N, 51, F)

## Discussion

The findings of this qualitative study illustrate how the experiences of health care providers during the simulated exercise helped change the nature of their relationships with their patients and highlighted the effect of simulation as a knowledge translation strategy. The exercise was able to deepen their learning, improve person-centredness and adherence counselling skills.

### Experiences of the simulated exercise

The experiences of health care providers were similar to those from a study in Swaziland, where adherence rates and experiences were compared to actual patients. The Swaziland study recognised many barriers to adherence, such as dosing device difficulties, forgetfulness and social life interruption, as in our study.^8^ Participants, especially those designated as caregivers, expressed excitement participating in this exercise and being able to administer liquid. Treatment supporters had mixed emotions about their role and felt that caregivers were sometimes not available to administer the placebo drug. A qualitative study from South Africa, exploring motivations, strategies and experiences of adult HIV patients, showed that any deviance from the normal daily routine resulted in forgetfulness.^16^ Some participants forgot to administer the medication because of either having other social commitments or forgetting and not being reminded by a treatment supporter. The responsibility of having to take care of a ‘child’ caused participants to be stressed and feel anxious. Their feelings were similar to a typical mother who faces multiple stressors, including scarce social support, stigmatisation and anxiety that her child may be HIV-positive.^17^ In real life, caregivers must deal with their own health, and psychosocial issues, besides being responsible for a child.^6^ A few participants struggled handling bottles and syringes for the first time during the exercise. They were aware that lay counsellors were usually responsible for demonstrating adherence to patients using a syringe, adaptor cap or a bottle.

Two participants identified that their own stigmatisation at work and sensitised them to the fact that their patients were facing the same challenges. Participants became increasingly aware of the importance of disclosing to members of a family living in the same household, after having experienced forms of stigma at the guesthouse, and while shopping. Our findings also supported the notion that failure to disclose a child’s status to household members interrupted their ability to administer ART.^18^ An ethnographic qualitative study conducted at a district hospital in Dar es Salaam, Tanzania, showed that having a treatment supporter or partners may combat HIV-related stigma.^19^

A few participants struggled to find the time to take the medication as agreed. Again, this was generally because of other social commitments or was reminded by their treatment supporters during the exercise. Often in real situations, the treatment support should develop strategies to address perceived lack of time to take the medication, either because of work or social issues within the family. These observations were similar to barriers identified in caregivers of children in large HIV treatment programs in Kenya, such as caregiver forgetfulness, problems with dose timing, and social issues like stigma.^20^

### Patient–provider relationship

One of the most advocated ways to improve adherence is to improve patient–provider relationships.^21^ Close friendships and connections were developed between participants during the exercise, which was a key intervention in adherence. Participants revealed that they were more aware of their patients’ circumstances because of their raised empathy levels during the exercise. This study highlighted the importance of communication to improve ART adherence in children. The relationship between a patient and a provider depends on the respectful treatment by both parties, which is central to the positive attitudes and behaviours of primary health care clinic staff.^22^ The research has also shown that there is a paradigm shift from a ‘detached paternalistic relationship towards a more empathetic, patient-centred model of care’.^23^ The relationship between caregivers and supporters is also very important and must be a highly trusting relationship.

### Adherence strategies

Many adherence strategies were identified during the exercise. Participants suggested using cell phone messaging (WhatsApp) as a medium to remind each other to administer V medication and to show evidence that administration had occurred. Text messaging, therefore, has the potential to improve adherence. This social media platform can ‘reduce the burden of disease on health care systems by rendering more efficient management, treatment, and education support’.^24^ The findings emphasise the importance of having treatment supporters to encourage adherence. The bonds that developed between participants made them realise the importance of buddy systems in their respective settings, which were often lacking. Even though the pairs were not related, they realised that nominating a supporter was a method of helping caregivers to manage their own illness. The participants agreed that the treatment supporter is someone with close links to the family, who is aware of his or her status and can support the caregiver and child once ART is initiated.^25,26^ Participants regarded intensive adherence counselling, which is normally done by lay counsellors, as a major strategy and felt confident in reinforcing this task. The participants realised that open-ended questions allowed patients to respond in a non-threatening way. Our findings support current literature, which shows that adherence counselling focusing on problem-solving may be effective in altering the behaviour of HIV patients, thereby improving adherence.^27^ This was an opinion agreed upon by all the participants.

## Limitations and strengths

There are a few limitations in this study. While the practical arrangement of administrating the placebo drug into sink is justifiable, it limits the authenticity of the simulation, as opposed to giving it to an actual child. The study was small, including 29 participants from across six provinces of South Africa. Inclusion criteria were specific for doctors and nurses working in government health care facilities managing HIV patients; therefore, care must be taken in transferring these results to other settings. The strength of the study was that FGDs were used to obtain detailed information about personal and group feelings. This was not the case prior to FGDs being introduced to the exercise.

## Recommendations

This study highlighted the challenges with dosing devices and packaging standards of paediatric ART. Future trainings should incorporate group-debriefing sessions to promote reflective learning, much as the focus groups did. Simple simulation exercises have the potential to improve adherence skills of health care providers in adherence and promote awareness of paediatric adherence challenges. Interventions like this and the application of such methods could also enhance learning of patient-centredness as a skill in teaching and learning. Even though this study suggested perceived improvements in clinical practice after a 3-month period, more evidence is needed to enhance understanding of the impact of this intervention on patient outcomes at a health systems level.

## Conclusion

The findings of our study focused on participants’ experiences of the simulated exercise, patient–provider relationships and adherence strategies. The study highlighted many paediatric ARV adherence issues, and health care providers felt empowered to more effectively counsel and clinically manage their patients. They were able to recognise barriers to adherence and interrogate their own relationships with their patients. Simulation is an effective tool to increase provider awareness and engagement around paediatric adherence issues.

## References

[1] World Health Organization, UNICEF, UNAIDS Global UpdateOn HIV Treatment 2013 : Results, impact and opportunities [homepage on the Internet]. WHO, UNICEF, UNAIDS, 2013 [cited 2018 Jan 02]. Available from: http://apps.who.int/iris/bitstream/10665/85326/1/9789241505734_eng.pdf

[2] Joint United Nations Programme on HIV/AIDS Ending AIDS: Progress towards the 90-90-90 targets. Geneva: UNAIDS; 2017.

[3] National Department of Health Clinical Guidelines for the Management of HIV & AIDS in Adults and Adolescents. South Africa 2010.

[4] Marchal B, Brouwere V De, Kegels G Viewpoint : HIV / AIDS and the health workforce crisis: What are the next steps ? Trop Med Int Heal. 2005;10(4):300–304. https://doi.org/10.1111/j.1365-3156.2005.01397.x 10.1111/j.1365-3156.2005.01397.x 15807792

[5] Davies MA, Keiser O, Technau K, Eley B, Rabie H, van Cutsem G Outcomes of the South African National Antiretroviral Treatment Programme for children: The IeDEA Southern African collaboration. SAMJ. 2009;99:730–737. 20128272 PMC2925142

[6] Pontali E Facilitating adherence to highly active antiretroviral therapy in children with HIV infection: What are the issues and what can be done? Paediatr Drugs. 2005;7(3):137–149. https://doi.org/10.2165/00148581-200507030-00001 15977960 10.2165/00148581-200507030-00002

[7] Müller AD, Jaspan HB, Myer L, et al Standard measures are inadequate to monitor pediatric adherence in a resource-limited setting. AIDS Behav. 2011;15(2):422–431. https://doi.org/10.1007/s10461-010-9825-6 20953692 10.1007/s10461-010-9825-6 PMC3032912

[8] Phelps BR, Hathcock SJ, Werdenberg J, Schutze GE Experiencing antiretroviral adherence: Helping healthcare staff better understand adherence to paediatric antiretrovirals J Int AIDS Soc [serial online]. BioMed Central Ltd; 2010;13(1):48 [cited 2015 Jul 01]. Available from: http://www.jiasociety.org/content/13/1/48 10.1186/1758-2652-13-48 PMC3004827 21134284

[9] Rudolph JW, Simon R, Rivard P, Dufresne RL, Raemer DB Debriefing with good judgment: Combining rigorous feedback with genuine inquiry. Anesthesiol Clin. 2007;25(2):361–376. https://doi.org/10.1016/j.anclin.2007.03.007 17574196 10.1016/j.anclin.2007.03.007

[10] Reisner SL, Mimiaga MJ, Skeer M, Perkovich B, Johnson CV, Safren SA A review of HIV antiretroviral adherence and intervention studies among HIV-infected youth. Top HIV Med. 2009;17(1):14–25. 19270345 PMC3752381

[11] Shear TD, Greenberg SB, Tokarczyk A Does training with human patient simulation translate to improved patient safety and outcome? Curr Opin Anaesthesiol [serial online]. 2013;26(2):159–163. [cited 2015 June 01]. Available from: http://www.ncbi.nlm.nih.gov/pubmed/23339975 10.1097/ACO.0b013e32835dc0af 23339975

[12] Chirowodza A, Green B, Mamdoo P, Du Toit C South to South Programme Work plan October, 2014–September 2015. Implementing Mechanism: HIV Innovations for Improved Patient Outcomes in South Africa. (Developing & Institutionalizing an Innovative Capacity Building Model to Support SA Government Priorities & to Improve HIV/TB Outcomes for Priority Populations) Cooperative Agreement No: AID-674-A-12-00031. South to South Programme for Comprehensive Family HIV Care & Treatment, Stellenbosch University, Cape Town, South Africa; 2014.

[13] Merriam-webster.com. Dictionary by Merriam-Webster: America’s most trusted online dictionary [homepage on the Internet]. 2018 [cited 2018 May 08]. Available from: https//www.merriam-webster.com/dictonary/empathy

[14] Raiman S, Michaels D, Nuttall J, Eley B A 4-pronged approach to addressing antiretroviral adherence in children: A paediatric pharmacy perspective. S Afr Pharmaceut J. 2008;75(8):18–21.

[15] Terre Blanche M, Durrheim K, Kelly K First steps in qualitative data analysis. University of Cape Town Publication; 2006, pp. 320–344.

[16] van Loggerenberg F, Gray D, Gengiah S, et al A qualitative study of patient motivation to Adhere to Combination Antiretroviral Therapy in South Africa. AIDS Patient Care STDS [serial online]. 2015;29(5):299–306. Available from: http://online.liebertpub.com/doi/10.1089/apc.2014.0293 10.1089/apc.2014.0293 PMC4410570 25692575

[17] Ickovics JR, Thayaparan B, Ethier KA Women and AIDS: A contextual analysis. In: Baum AS, Revenson TA, Singer JE, editors Handbook of health psychology. New Jersey: Lawrence Erlbaum Associates; 2001; p. 817–839.

[18] Coetzee B, Kagee A, Bland R Barriers and facilitators to paediatric adherence to antiretroviral therapy in rural South Africa: A multi-stakeholder perspective. AIDS Care [serial online]. 2014;27(3):315 Available from: http://www.tandfonline.com/doi/abs/10.1080/09540121.2014.967658 10.1080/09540121.2014.967658 PMC4305492 25355176

[19] O’Laughlin KN, Wyatt MA, Kaaya S, Bangsberg DR, Ware NC How treatment partners help: Social analysis of an African Adherence Support Intervention. AIDS Behav [serial online]. 2012;16(5):1308–1315. Available from: http://link.springer.com/10.1007/s10461-011-0038-4 10.1007/s10461-011-0038-4 PMC3354325 21947835

[20] Vreeman RC, Nyandiko WM, Liu H, et al Comprehensive evaluation of caregiver-reported antiretroviral therapy adherence for HIV-Infected Children. AIDS Behav [serial online]. 2015;19(4):626–634. Available from: http://doi:10.1007/s10461-015-0998 10.1007/s10461-015-0998-x PMC4393778 25613594

[21] Vermiere E, Hearnshaw H Patient adherence to treatment: Three decades of research. A comprehensive review. J Clin Pharm Therapeut. 2001;26:331–342. https://doi.org/10.1046/j.1365-2710.2001.00363.x 10.1046/j.1365-2710.2001.00363.x 11679023

[22] Gilson L, Palmer N, Schneider H Trust and health worker performance: Exploring a conceptual framework using South African evidence. Soc Sci Med [serial online]. 2005;61(7):1418–1429. [cited 2015 Oct 08]. Available from: http://linkinghub.elsevier.com/retrieve/pii/S027795360400646X 10.1016/j.socscimed.2004.11.062 16005777

[23] Haas J, Shaffir W The professionalization of medical students: Developing competence and a cloak of competence. Symbolic Interact. 1977;1:71–88. https://doi.org/10.1525/si.1977.1.1.71

[24] Devi BR, Syed-Abdul S, Kumar A, et al mHealth: An updated systematic review with a focus on HIV/AIDS and tuberculosis long term management using mobile phones Comput Meth Programs Biomed [serial online]. Elsevier Ireland Ltd; 2015;1–9. [cited 2015 Sep 13]. Available from: http://linkinghub.elsevier.com/retrieve/pii/S016926071500200X 10.1016/j.cmpb.2015.08.003 26304621

[25] Ware NC, Idoko J, Kaaya S, et al Explaining adherence success in Sub-Saharan Africa: An ethnographic study. PLoS Med [serial online]. 2009;6(1):e11 [cited 2015 Oct 05]. Available from: http://www.plosmedicine.org/article/info:doi/10.1371/journal.pmed.1000011 10.1371/journal.pmed.1000011 PMC2631046 19175285

[26] Wouters E, Van Damme W, Van Loon F, van Rensburg D, Meulemans H Public-sector ART in the Free State Province, South Africa: Community support as an important determinant of outcome Soc Sci Med [serial online]. Elsevier Ltd; 2009;69(8):1177–1185. [cited 2015 Oct 05]. Available from: http://linkinghub.elsevier.com/retrieve/pii/S0277953609004912 10.1016/j.socscimed.2009.07.034 19692165

[27] Dewing S, Mathews C, Schaay N, Cloete A, Simbayi L, Louw J Improving the counselling skills of lay counsellors in antiretroviral adherence settings: A cluster randomised controlled trial in the Western Cape, South Africa. AIDS Behav. 2015;19(1):157–165. 24770948 10.1007/s10461-014-0781-4

